# Plant-Derived Polysaccharides Regulated Immune Status, Gut Health and Microbiota of Broilers: A Review

**DOI:** 10.3389/fvets.2021.791371

**Published:** 2022-01-28

**Authors:** Bolin Zhang, Ning Liu, Meilin Hao, Jianhong Zhou, Yuxiao Xie, Zhen He

**Affiliations:** Department of Biology and Agriculture, Zunyi Normal College, Characteristic Laboratory of Animal Resources Conservation and Utilization of Chishui River Basin, Zunyi, China

**Keywords:** polysaccharide, intestinal morphology, immunity, gut microbiota, broilers

## Abstract

In modern intensive breeding system, broilers are exposed to various challenges, such as diet changes and pathological environment, which may cause the increase in the incidence rate and even death. It is necessary to take measures to prevent diseases and maintain optimal health and productivity of broilers. With the forbidden use of antibiotics in animal feed, polysaccharides from plants have attracted much attention owing to their lower toxicity, lower drug resistance, fewer side effects, and broad-spectrum antibacterial activity. It had been demonstrated that polysaccharides derived from plant exerted various functions, such as growth promotion, anti-inflammation, maintaining the integrity of intestinal mucosa, and regulation of intestinal microbiota. Therefore, the current review aimed to provide an overview of the recent advances in the impacts of plant-derived polysaccharides on anti-inflammation, gut health, and intestinal microbiota community of broilers in order to provide a reference for further study on maintaining the integrity of intestinal structure and function, and the related mechanism involved in the polysaccharide administration intervention.

## Introduction

In modern breeding system, broilers are often exposed to various external factors, such as diseases, nutritional, and environmental challenges, which lead to impaired growth, poor health, and even high mortality percentages owing to their imperfect intestinal function development and immature immunity (1). In addition, the pathogenic bacteria existing in the breeding environment can cause diseases influencing the health of broilers, which in turn results in high mortality rates and huge economic losses in the poultry industry (2). In the past, antibiotics were widely used as performance-enhancing feed additives in poultry production to promote productivity and prevent diseases (3). However, the extensive use of antibiotics not only caused the problems of antibiotic resistance but also induced the accumulation of antibiotic residues in animal products, and developed new strains of drug-resistant pathogenic bacteria (4). Therefore, the addition of antibiotics in animal feed was forbidden in most countries of the world. It has become imperative to find safe and effective alternatives to antibiotics that can improve chicken health by improving the gut health and maintaining the optimum growth of broilers.

Polysaccharides are large molecular weight polymers and bioactive macromolecules with complex molecular structures, which are usually composed of more than 10 monosaccharides linked by glycosidic linkages in linear or branched chains (5, 6). In general, polysaccharides are composed of complex sets of monosaccharides, such as mannose, galactose, glucose, and arabinose. Besides, it is known that the structural features of polysaccharides, the glycosidic bond of the main chain and molar mass, are related to their biological properties (7). Moreover, the bioactivities of polysaccharides could be enhanced by moderate structural modification (8, 9). Plants are an important source of natural polysaccharides. With regard to their different sources, plant polysaccharides are classified into dietary fiber (inulin, gums, pectin), algae, and traditional herbs (10). Numerous studies have been conducted to investigate the effects of bioactive ingredients derived from plant, such as polysaccharide, which was used as growth promoter feed additives to enhance the overall performance parameters as well as health conditions of broilers owing to their safety properties, including lower drug resistances and relatively fewer side effects (11, 12). It has been documented that polysaccharides extracted from plants exhibited promising effects on anti-inflammation, modulation of gut health, and intestinal microbiota of broilers (13–16). Therefore, in the current review, we attempted to summarize the various bioactive effects of plant polysaccharides on growth performance, immune status, gut health, and microbiota of broilers.

## Performance Productivity

**Figure 1:**
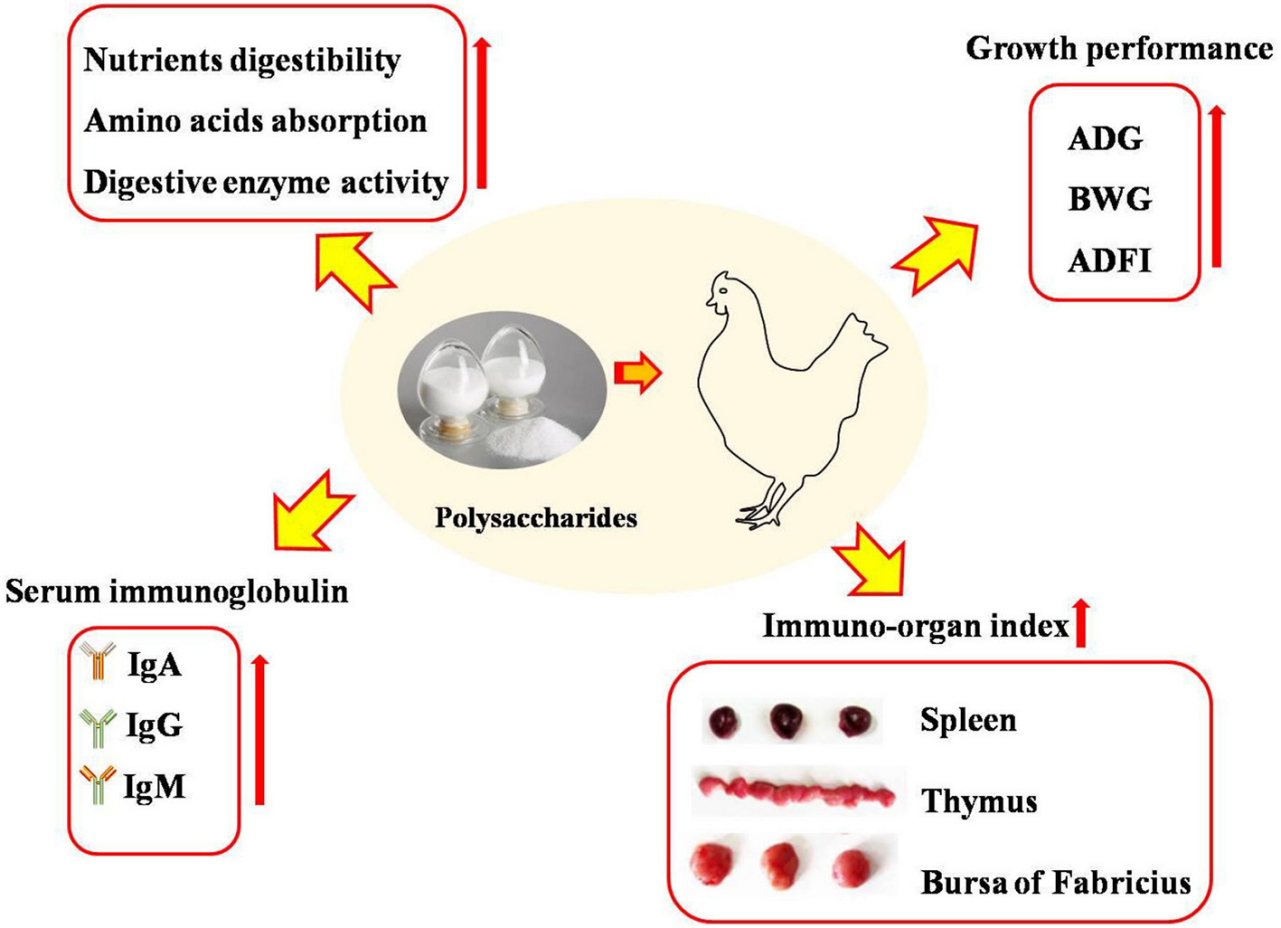
Dietary polysaccharides supplementation improved growth performance, increased nutrients digestibility and amino acids absorption, elevated the content of serum immunoglobulin and immune-ogran index of broilers. IgA, immunoglobulin A; IgG, immunoglobulin G; IgM, immunoglobulin M; ADG, average daily weight gain; BWG, body weight gain; ADFI, average daily feed intake.

In previous studies, it has been demonstrated that polysaccharides had the potential to enhance the growth performance of broilers (17, 18) (Figure 1). It was reported that higher-dose polysaccharides supplemented in diets were beneficial to promote growth performance, while the effects of lower-dose polysaccharides were of no significance (19). Wu (17) suggested that a diet supplemented with 500, 1,000, and 2,000 mg/kg of *Astragalus membranaceus* polysaccharide (*APS*) effectively enhanced the body weight gain of broilers after a 6-week feeding trial. In contrast, Chen et al. (20) demonstrated that 200 mg/kg of *APS* polysaccharide supplementation did not affect the growth performance of broilers. The results mentioned above indicated that the effects of polysaccharides used as prebiotics and growth-promoter ingredients on the growth performance of broilers were in dose-dependent manners. The growth-promoting effects of polysaccharides may be due to its stimulating effects on the activities of digestive enzymes (17, 18), the promotion of nutrient digestibility (21), and the absorption of amino acids (22).

In previous studies, it has been demonstrated that broilers in the status of immune suppression showed depressed growth performance (9, 23), as evidenced by lower weight gain and higher ratio of feed intake to weight gain, but dietary polysaccharide supplementation could be of benefit in reversing the negative effects on growth performance of broilers by inducing immune stimulation. Dietary *Achyranthes bidentata* polysaccharide supplementation increased body weight and average daily weight gain of broilers challenged with *Escherichia coli* K88 (14). It was also reported that *Salmonella* serotype Enteritidis-infected broilers exhibited impaired growth performance, but this was relieved by diets supplemented with polysaccharides isolated from alfalfa (24). Additionally, the biological activities of polysaccharides are closely related to their molecular weight and specific spatial structures (25). It has been demonstrated that, compared with untreated *APS*, administration of sulfated *APS* or γ-irradiated *APS* in diets was more effective in improving growth performance of broilers under the immunosuppression status (8, 9, 16). The results mentioned above indicated that polysaccharides or structurally modified polysaccharides were able to alleviate the negative effects on growth performance induced by bacterial infection.

Different from the beneficial effects of polysaccharides derived from traditional herbs and alfalfa on growth performance of broilers in normal or immunosuppression status, studies about diets supplemented with polysaccharides from dietary fiber (inulin, gum, and pectin) had no effects or even negative effects on growth performance of broilers. Ortiz et al. (26) suggested that the inclusion of inulin in diets exerted no growth-promoting effect at graded concentrations from 5 to 20 g/kg. Similarly, it was reported that incorporating inulin into a diet at 1, 2, and 4% dose did not improve the Overall growth performance of broilers (27). However, the inclusion of gum and pectin in diets had negative effects on growth performance of broilers, as evidenced by the depressed weight gain, lower feed intake, and higher ratio of daily weight gain to feed intake (28, 29), which maybe directly linked with the increased intestinal viscosity owing to the intake of indigestible polysaccharide such as pectin and gum (30).

## Immnune Organ and Serum Immunoglobulin

The development of immune tissues is the basis of immune functionality, and the development and maturation of lymphocytes is often used as an indicator of immunity (Figure 1). Larger organ weight and index implies stronger humoral and cellular immune capacity (31). It was reported in a previous study that *APS* and γ-irradiated *APS* supplementation increased the relative weight of the thymus and spleen of broilers, as well as promoted the proliferation of T lymphocytes (9). Similarly, Chen et al. (20) suggested that *APS* had significant immune-stimulating effects on splenocyte proliferation. Besides, γ-irradiated *APS*, not *APS*, enhanced the proliferation of B lymphocytes, indicating that γ-irradiated *APS* was more effective than *APS* in immune modulation (9). The reason for the higher activity of γ-irradiated *APS* was that irradiation leads to the cleavage of glycosidic bonds by electromagnetic waves of gamma rays and resulted in low molecular weight polysaccharide products, which affected their biological activities (9, 32, 33). *Enteromorpha* polysaccharide administration increased the relative weight of the bursa of Fabricius in broilers (34) and improved the morphology of the bursa of Fabricius, including infiltration of inflammatory cells, destruction of the cell structure, and cell necrosis (35). It has been demonstrated that *Camellia oleifera* cake polysaccharides increased the weight or index of the spleen and thymus of broilers (19). Polysaccharides were also benefited to improve the T-lymphocyte percentage and enhance the development of immune organs, evidenced by the increased lymph follicle area in the bursa of Fabricius and white pulp area expansion, which suggested that polysaccharide treatment elicited lymphocyte formation in the bursa of Fabricius (36). Further study by transcriptome analysis revealed that dietary *Enteromorpha* polysaccharides regulated 20 differentially expressed genes of the bursa of Fabricius, which were mainly enriched in negative regulation of the Toll-like receptor signaling pathway and mainly enriched in phagosome, mitophagy-animal, *Salmonella* infection, and autophagy signaling pathways (34). Moreover, it was identified that algae-derived polysaccharides ameliorated the impairment of the bursa of Fabricius in heat-stressed broilers via modulation of the NF-κB signaling pathway (35). Guo et al. (37) demonstrated that dietary *marine algal* polysaccharide administration alleviated damage and apoptosis of the bursa of Fabricius in broilers induced by aflatoxin B1, which might be associated with p38MAPK-Nrf2/HO-1 and mitochondrial apoptotic signaling pathways.

Polysaccharides derived from plants could activate macrophages by recognizing and binding to specific receptors on the surfaces of macrophages, which is beneficial in improving immune function and exerting an immunomodulatory effect (38). Serum immunoglobulin (Ig), such as IgA, IgG, and IgM, play important roles in the immune system in poultry (39) and are related to the state of the immune function (Figure 1). It was reported that *Lycium barbarum* polysaccharide supplementation contributed to enhancing the concentrations of IgA, IgG, and IgM in the serum (18). Wu et al. (40) reported that inulin addition resulted in more IL-6 and tended to increase the concentration of IgA and IgM. Wu (17) and Long et al. (41) suggested that *Astragalus membranaceus* polysaccharides or *Acanthopanax senticosus* polysaccharide supplementation promoted the humoral immunity of broilers as evidenced by the increased concentration of IgA and IgG in the serum. The information mentioned above confirmed that polysaccharides could be beneficial in improving the humoral immunity of broilers.

## Inflammation Regulation

**Figure 2:**
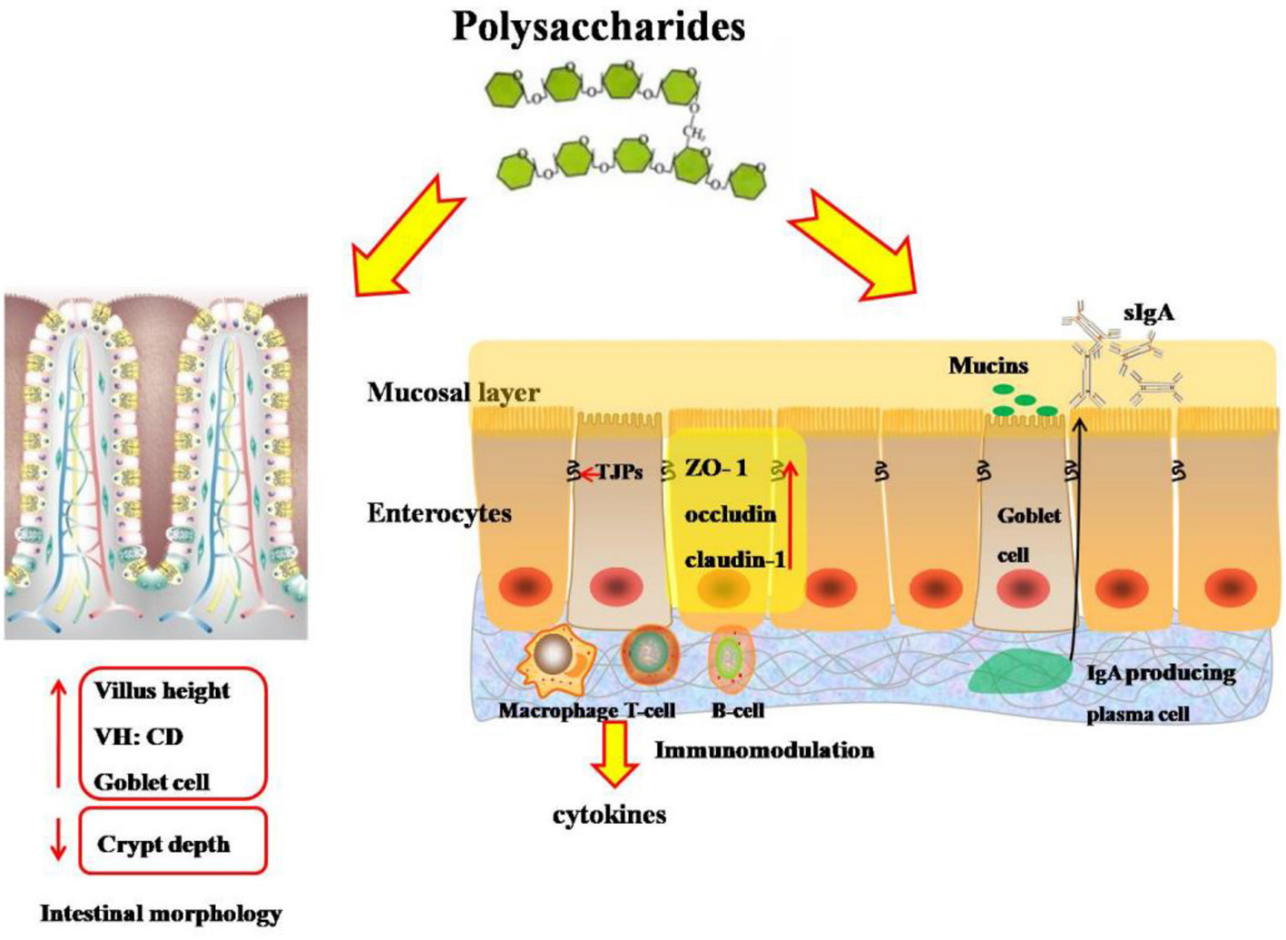
Effects of polysaccharides on intestinal morphology and intestinal mucosal immunity and barrier integrity of broilers. VH:CD, the ratio of villus height to crypt depth; TJPs, tight junction proteins; ZO-1, zonula-1; T-cell, T lymphocyte; B-cell, B lymphocyte; IgA, immunological A; sIgA, secretory immunological A.

Polysaccharides, a kind of natural macromolecular polymers, have attracted extensive attractions due to their diverse and important biological activities in immunomodulatory effects and anti-inflammation (6, 9, 13). Polysaccharides could be an immunopotentiator to promote the proliferation of B lymphocytes and T lymphocytes, the increase in serum antibody titer, and the reduction in the ratio of blood heterophil to lymphocyte (9, 12) (Figure 2). CD3+, CD4+, and CD8+ are vital T-lymphocyte markers and can be used as an indicator of the T-cell response (13). *Astragalus* polysaccharide increased CD3+ and CD8+ T-cell populations, as well as the numbers of CD4+ lymphocytes (13), indicating that *APS* administration benefited in cellular immunity.

In addition, cytokines are important mediators of immune responses. Intraepithelial lymphocytes, which are immunocompetent cells that first encounter enteric pathogens invading the mucosa and play an important role in gut mucosal immunity, can program cytokine production (such as IL-2 and IFN-γ) to protect the intestines from bacterial and viral invasion (8). Li et al. (42) found that γ-irradiated *APS* enhanced the numbers of intraepithelial lymphocytes, as well as increased mRNA expressions of IL-2 and IFN-γ. Moreover, it was demonstrated that dietary polysaccharide supplementation could modulate immune functions through regulating the production of cytokines and proinflammatory mediators. Zhang et al. (43) suggested that *Glycyrrhiza* polysaccharide mitigated the LPS-induced increase in IL-1β and IFN-γ in the liver. Liu et al. (44) found that *algae-*derived polysaccharide supplementation lowered the relative mRNA expression of TNF-α and IL-1β in the duodenum of broilers that suffered from heat stress. In addition, structural modification contributed to boost the activity of the polysaccharides. Wang et al. (12, 45) suggested that, in comparison with untreated *APS*, sulfated *APS* exhibited more effective anti-inflammatory effect *in vitro* and *in vivo*, evidenced by the downregulation of the increased expression of TNF-α and IL-1β induced by the LPS challenge. Similarly, Li et al. (9) and Ren et al. (32) demonstrated that γ-irradiated administration improved the immunomodulating activity of *APS*, which increased the intestinal intraepithelial lymphocytes and mRNA expressions of IL-2 and IL-10 in the jejunum of broilers. From the information mentioned above, it can be concluded that polysaccharides can promote the proliferation of immune cells and exert anti-inflammatory activity. Therefore, it is necessary to look for and evaluate polysaccharides with immunostimulating properties and further expand their application in the breeding of broilers.

## Gut Health and Intestinal Muscosal Barrier

The intestine is responsible for nutrient digestion and absorption, and the growth performance of broilers is associated with the morphological and functional integrity of the digestive system (46) (Figure 2). The values of villus height (VH) and the ratio of villus height to crypt depth (VH:CD) of the small intestine are useful indicators to estimate the absorption capacity of the small intestine (47). After 35 days of feeding supplemented with algae-derived polysaccharides from *Enteromorpha*, it was found that the VH and villus surface area of the duodenum and ileum were both increased (35). Previous studies also demonstrated that broilers fed with diets containing 600 mg/kg of *Astragalus* polysaccharides had higher VH and ratio of VH:CD (1, 42), suggesting that polysaccharides contributed to improve intestinal morphology associated with the prebiotic function of polysaccharides. Polysaccharides could work as a substrate on microflora population and bacterial metabolites, and aids in stimulating the fermentation rate and increasing the production of short-chain fatty acids, which benefits the differentiation and proliferation of intestinal epithelial cells (35, 48). Interestingly, *APS* supplementation on intestinal morphology showed transgenerational effect. The paternal dietary *APS* supplementation in a dose of 10 g/kg could promote transgenerational intestinal morphology of broiler chickens (49).

The paracellular permeability of the intestinal barrier is regulated by a complex protein system called tight junctions (50) (Figure 2). Tight junctions, the multiprotein complexes, are composed of transmembrane proteins, peripheral membrane proteins, and regulatory molecules including kinases, among which the claudin family proteins are the most important of the transmembrane proteins, whereas the ZO family proteins are the peripheral membrane proteins and are crucial to tight junction assembly (51). D-lactic acid and diamine oxidase are generally used as sensitive biomarkers for intestinal permeability to reflect the extent of damage in the intestine tract (52). Polysaccharide supplementation decreased the content of D-lactic acid and the activity of diamine oxidase in the serum, implying that polysaccharides improved the intestinal permeability and protected the intestinal barrier function (35). Accordingly, accumulated evidences showed that polysaccharides improved the intestinal integrity, maintained the integrity of intestinal epithelium, and upregulated mRNA expressions or protein abundances of tight junction proteins such as ZO-1, occludin, and claudin-1 in the jejunum and ileum of broilers in normal or stressed conditions (12, 24, 44). It was generally reported that polysaccharides could not be absorbed through the intestinal mucosa into the body, so the health benefits of polysaccharides were rooted in their effects on the intestinal mucosal immune system (49). Therefore, the reasons for maintaining the integrity of intestinal mucosa by polysaccharides could be speculated as follows: First, polysaccharides improved the proliferation and maturation of intestinal epithelial cells to regulate the intestinal immune functions by synthesizing and releasing mucins, and enhancing the integrity of intestinal mucosa (42, 53). Second, a diet supplemented with polysaccharides is beneficial in promoting the development of IgA-producing cells in the intestinal mucosa and increasing the production of secretory immunoglobulin A, which is a major antibody isotype in the intestinal mucosal immunity that can prevent pathogens and toxins from invading the epithelial surface, and maintain the homeostasis of the intestine (24, 42, 54). Additionally, the protective effects of polysaccharides on the intestinal mucosa barrier were partly associated with its anti-inflammatory effects (12, 55).

## Gut Microbial

The intestines are populated by a complex and dynamic microbial community, which contributes to the health status of the host animals and is the first barrier against pathogens (41, 56). The cecum is often the focus for chicken gut microbial studies because almost microbial populations are colonizations in this site and also due to their role in carbohydrate fermentation, which is considered to contribute significantly to general intestinal “health” (57). Through 12 phyla of bacteria, represented analysis by high-quality 16S rRNA gene sequences of chicken bacteria, the predominant bacteria were *Firmicutes* (almost 70% of sequences) and *Bacteroidetes* (12.3% of sequences) phyla, with cell densities exceeding 10^11^ per g of digesta (58, 59). Within the phylum *Firmicutes, Clostridium, Ruminococcus, Lactobacillus, Eubacterium, and Fecalibacterium* are the domain bacteria (60), while genera *Bacteroides, Prevotella, Parabacteroides*, and *Alistipes* are the domain bacteria in phylum *Bacteroidetes* (58). However, the composition of the intestinal microbiota can be altered by dietary manipulations and pathological environment (61).

**Figure 3:**
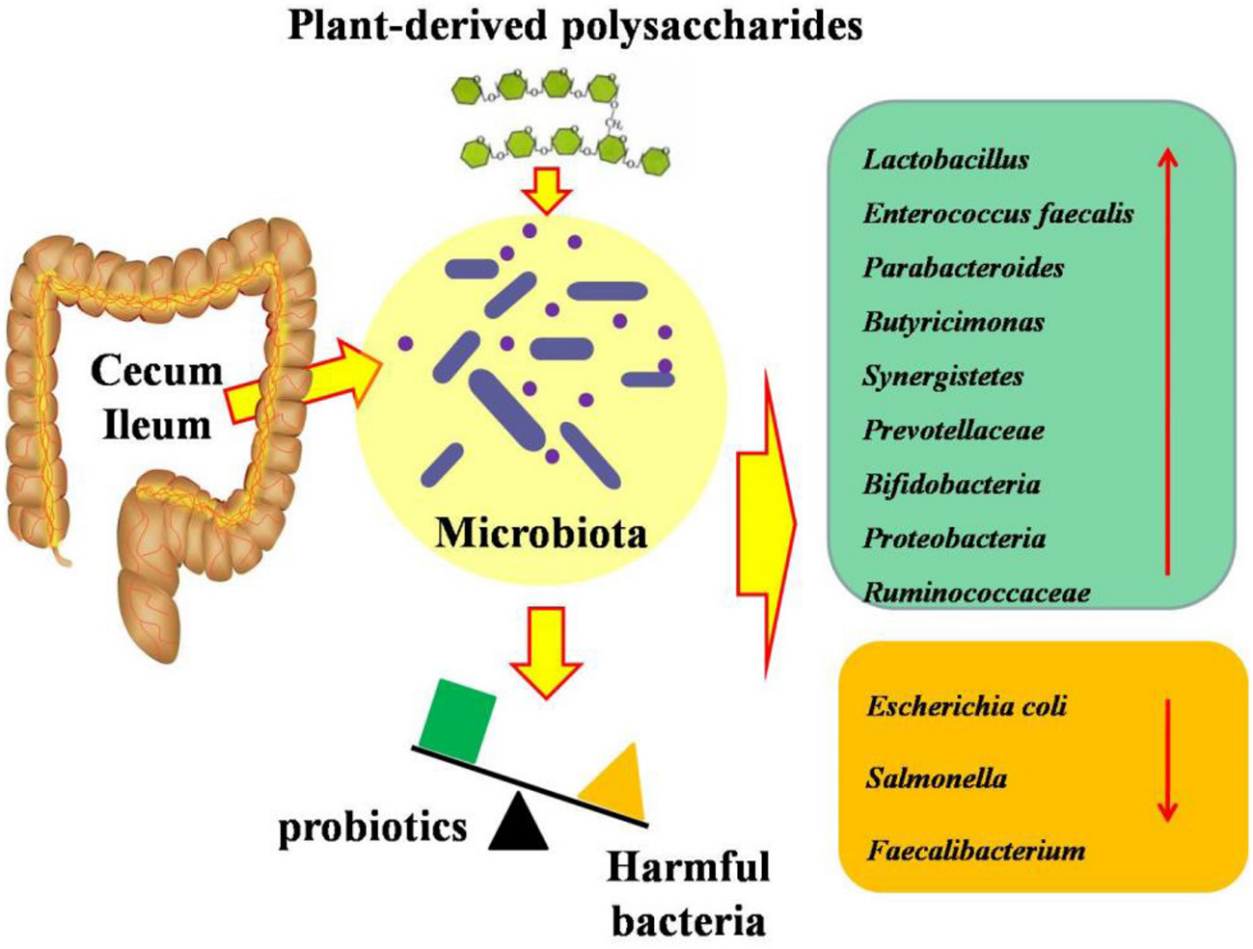
Effects of plant-derived polysaccharides on intestinal microbiota in ileum and cecum of broilers.

**Figure 4:**
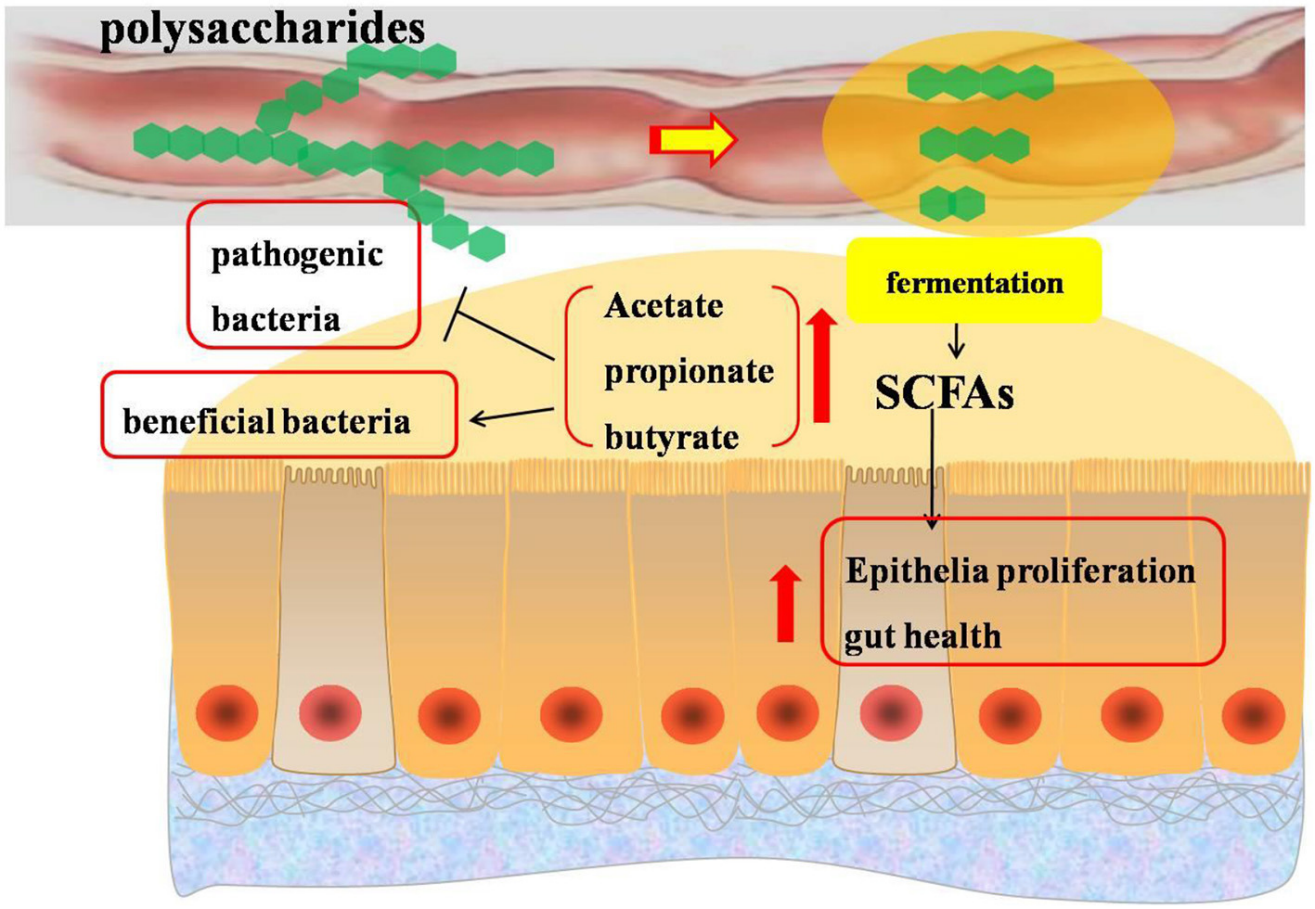
Effects of polysaccharides on short-chain fatty acids in the intestine of broilers. SCFAs, short-chain fatty acids.

It has been demonstrated that plant bioactive substances can be fermented to produce metabolites by the gut microbiota in the distal gastrointestine, and further change and reshape the intestinal microbial community via fermentation (62) (Figures 3, 4). Polysaccharides, serving as unique carbon sources for intestinal bacteria, have a beneficial microbial modulation effect, which demonstrated that broilers receiving polysaccharides have a higher abundance of beneficial bacteria and a lower abundance of harmful bacteria (36). *Camellia oleifera cake* polysaccharide supplementation could promote the growth of probiotics and had the potential to inhibit the number of pathogenic bacteria (19). Numerous studies proved that polysaccharide (such as *Achyranthes bidentata* polysaccharides, *Camellia oleifera* cake polysaccharides, *APS*, and *Acanthopanax senticosus* polysaccharides) treatment increased the abundances of *Proteobacteria, Bacteroides, Lactobacillus*, and *Ruminococcaceae*, and decreased the abundances of *Escherichia coli* and *Salmonella* (14, 19, 36, 41). It was reported that inulin supplementation decreased the cecal concentrations of *Escherichia coli, Salmonella*, and *Campylobacte*r in broilers (63), and increased *Bifidobacterium* (64). Similarly, Xia et al. (27) found that there was a decrease in *Firmicutes and Actinobacteria*, and an increase in *Bacteroidetes* and *Proteobacteria* in response to inulin inclusion. Wu et al. (40) suggested that inulin supplementation decreased the numbers of *Escherichia coli* and pH in the cecum of broilers. The changes in these bacteria contributed to reduce intestinal pH, improve the intestinal microbial balance via reducing the population of pathogenic species, and thus improves the health of the host (65). However, pectin and gum addition significantly increased intestinal viscosity, which further influenced microbial activity. It has been demonstrated that pectin administration significantly increased the contribution of anaerobic bacteria, affected the microbial composition in the small intestine, particularly that of *Enterococci, Clostridia*, and *Escherichia coli*, which may be responsible for the lower antinutritive properties induced by pectin supplementation (28).

Previous studies suggested that certain gut bacteria were responsible for the breakdown of polysaccharides not metabolized during transit through the small intestine into short-chain fatty acids, mainly including acetate, propionate, and butyrate, which contributed to promote epithelial proliferation and affect the intestinal ecology of the host animals (62, 66). The increased concentration of short-chain fatty acids in the gastrointestine is associated with an increase in the population of beneficial bacteria and a decrease in pathogenic bacteria (67, 68). The phylum *Firmicutes* (genus *Faecalibacterium, Shuttleworthia*, and *Ruminococcaceae*) and *Bacteroidetes* were known for their fermentative end products in the form of different types of short-chain fatty acids, such as acetate, butyrate, and lactate (69). *Parabacteroides distasonis*, a beneficial bacterium in the bowel, could regulate host metabolism and ameliorate metabolic dysfunction by producing succinate (70). Similarly, *Butyricimonas* bacteria could improve intestinal barrier functions by producing short-chain fatty acids from the fermentation of polysaccharides such as alfalfa polysaccharide (71). The information mentioned above indicated that polysaccharides could influence gut microbial community composition and contribute to produce short-chain fatty acids to provide energy for enterocytes and improve gut health (72). Besides, short-chain fatty acids might inhibit *Salmonella* growth and invasion (73) and reduce the abundance of *Escherichia coli*, which might be associated with the increased acidity in the cecum induced by the production of short-chain fatty acids (74).

## Conclusion and Future Prospects

Plant-derived polysaccharides are vital ingredients with multifunctions. The application of polysaccharides in poultry breeding exhibited more advantages, such as low drug resistance, low side effects, and broad-spectrum antibacterial effect. Polysaccharides from plants are beneficial for growth promotion and proliferation of intestinal epithelial cells, activating immune system, regulating the abundance of intestinal microbiota, and protecting the immune barrier of the intestinal mucosa in broilers. However, there are still some questions about polysaccharides applied in broiler diets to be clarified. First, plant polysaccharides are a class of polymeric molecules composed of long chains of monosaccharide units bound together by glycosidic linkages. Therefore, some attention should be paid to ascertain the exact effective ingredients of polysaccharides. Second, the relationship of the structure and activity of polysaccharides needs further study. Third, the evaluation criteria for the efficiency of polysaccharides are insufficient. Fourth, the regulation mechanism of polysaccharides on intestinal microbiota is still completely unknown and the combined strategies are needed to clarify it. In addition, the combined application of plant polysaccharides with some natural active substances to maintain gut health and the optimal growth performance of broilers may be in the right direction and can contribute to explore more biologically active plant-derived polysaccharides.

## Author Contributions

BZ wrote this manuscript. NL, YX, and ZH helped in literature search. MH, NL, and JZ assisted in revising the manuscript. All authors contributed to the article and approved the submitted version.

## Funding

This work was supported by tBasic Project of Guizhou Provincial Natural Science Foundation [Qian Kehe (2017) 1205], Zunyi 15851 Talent Project (2050020213#), Zunyi City- School Joint Fund (Zunshi Kehe No. 277) and Zunshi Kehe (2018) No. 08], Rural Industrial Revolution Project of Guizhou Province (Zunshi He Rural Industry 201905), Zunyi City-School Joint Fund (Zunshi Kehe HZ 277), and Characteristic Laboratory of Animal Resources Conservation and Utilization of Chishui River Basin(Qianjiaohe KY [2013]111-03).

## Conflict of Interest

The authors declare that the research was conducted in the absence of any commercial or financial relationships that could be construed as a potential conflict of interest.

## Publisher's Note

All claims expressed in this article are solely those of the authors and do not necessarily represent those of their affiliated organizations, or those of the publisher, the editors and the reviewers. Any product that may be evaluated in this article, or claim that may be made by its manufacturer, is not guaranteed or endorsed by the publisher.
